# Epidemiologic Differences Between Cyclosporiasis and Cryptosporidiosis in Peruvian Children

**DOI:** 10.3201/eid0806.01-0331

**Published:** 2002-06

**Authors:** Caryn Bern, Ynes Ortega, William Checkley, Jacquelin M. Roberts, Andres G. Lescano, Lilia Cabrera, Manuela Verastegui, Robert E. Black, Charles Sterling, Robert H. Gilman

**Affiliations:** * Centers for Disease Control and Prevention, Atlanta, Georgia, USA; † University of Georgia, Griffin, Georgia, USA; ‡ Asociación Benéfica PRISMA, Lima, Peru; § Johns Hopkins University, Baltimore, Maryland, USA; ¶ Universidad Peruana Cayetano Heredia, Lima, Peru; and #University of Arizona, Tucson, Arizona, USA

**Keywords:** Cryptosporidiosis/epidemiology, cyclosporiasis/epidemiology, risk factors, Peru/epidemiology, intestinal diseases, parasitic/epidemiology/etiology

## Abstract

We compared the epidemiologic characteristics of cyclosporiasis and cryptosporidiosis in data from a cohort study of diarrhea in a periurban community near Lima, Peru. Children had an average of 0.20 episodes of cyclosporiasis/year and 0.22 episodes of cryptosporidiosis/year of follow-up. The incidence of cryptosporidiosis peaked at 0.42 for 1-year-old children and declined to 0.06 episodes/child-year for 5- to 9-year-old children. In contrast, the incidence of cyclosporiasis was fairly constant among 1- to 9-year-old children (0.21 to 0.28 episodes/child-year). Likelihood of diarrhea decreased significantly with each episode of cyclosporiasis; for cryptosporidiosis, this trend was not statistically significant. Both infections were more frequent during the warm season (December to May) than the cooler season (June to November). Cryptosporidiosis was more frequent in children from houses without a latrine or toilet. Cyclosporiasis was associated with ownership of domestic animals, especially birds, guinea pigs, and rabbits.

The coccidian protozoal parasites *Cyclospora cayetanensis* and *Cryptosporidium* *parvum* are recognized diarrheal pathogens among children in developing countries (1–4), but longitudinal data, especially for cyclosporiasis, are sparse. *Cyclospora cayetanensis* is more closely related genetically to *Eimeria* species than to *Cryptosporidium* species (5), and the two organisms have biological differences. For example, *C. parvum* is infectious when excreted and can be transmitted directly from person to person; *Cyclospora cayetanensis* requires a period of time in the environment to sporulate into the infectious form (3), decreasing the likelihood of direct person-to-person spread. *Cryptosporidium parvum* infects both humans and a variety of mammals (6), and evidence is mounting that non-*parvum* zoonotic *Cryptosporidium* species can also infect immunocompetent humans (7,8). Conversely, natural or experimental infection of animals with *Cyclospora cayetanensis* has not been convincingly demonstrated (9–11). Thus, cryptosporidiosis is transmitted through a variety of routes, including contaminated water or food, from person to person, or from animal to person. In contrast, the only major known risk factors for cyclosporiasis are consumption of contaminated water or produce (12–14).

Surveillance data suggest that both organisms are associated with diarrheal illness and asymptomatic infection but differ in their seasonality and susceptible age groups (15). The reasons for these differences are not well understood. Cohort studies of children in Peru provided an opportunity to better understand the characteristics of endemic cryptosporidiosis and cyclosporiasis. The objectives of the analysis were to provide a detailed description of the longitudinal epidemiology of the two organisms and to seek risk factors for infection.

## Materials and Methods

### Study Participants

Field work was conducted in the periurban *pueblo joven* (shantytown) of Pampas de San Juan de Miraflores, 25 km from the center of Lima, Peru. In the 1980s this community (pop. approximately 40,000) was heavily settled by immigrants from rural areas. Immigration to the community has slowed, and general living conditions have improved. In 1995, 97% of houses had electricity, 48% had toilets, and 64% had a household water connection (Asociación Benéfica PRISMA, Lima, Peru, unpub. data, 1995).

Our analysis was based on longitudinal data from two cohort studies conducted simultaneously from February 1995 to December 1998. The birth cohort study included all children born during the recruitment period whose mothers consented to participate; its major objectives were to elucidate the relationship between diarrheal disease and nutritional status (16) and to study the epidemiology of viral gastroenteritis. The objective of the other cohort study was to examine the epidemiology of cyclosporiasis. Children from 1 month to 10 years of age were chosen at random from the complete census of the community. Siblings of birth cohort children could be enrolled in the cyclosporiasis cohort if they were chosen by random selection. Twenty sibling pairs, a number too small to allow analysis of household clustering, were included in the analysis. Excluding at random one member of each sibling pair had no effect on results, and both siblings are included in the analysis presented here.

The same epidemiologic data and specimens were collected from children in both cohort studies. At the time of recruitment, field workers collected data regarding household characteristics, including type of housing, sanitary facilities, water source, and presence of animals. Field workers visited each household daily throughout the follow-up period to compile a daily record of the presence or absence of diarrhea in the child in the primary caretaker’s opinion, number of bowel movements, and consistency of stools (liquid, semiliquid, or formed).

Stool specimens were collected weekly from all children, on the first day of a diarrheal episode, and, when one of the pathogens of interest was detected, daily until negative. Stool specimens were transported without preservative and arrived in the laboratory within 24 hours of collection. Each specimen was processed by a standard ether concentration procedure and examined microscopically for *Cryptosporidium* species on modified acid-fast Ziehl-Neelsen stained slides and for *Cyclospora* *cayetanensis* on wet mount by direct examination and epifluorescence (17,18) in the pathology laboratory of the Universidad Peruana Cayetano Heredia.

### Epidemiologic Analysis

We defined a day with diarrhea as a 24-hour period during which the child was reported to have three or more liquid or semiliquid stools and, in addition, was thought by his or her primary caretaker to have diarrhea. An episode of diarrhea was considered to end when the child had at least 3 consecutive days that did not meet the criteria for a day with diarrhea. An episode of cryptosporidiosis or cyclosporiasis was defined by one or more stool specimens positive for the respective parasite. An episode of infection was considered to end on the last day of oocyst detection, followed by at least three negative stools and no oocyst detection for at least 28 days. An episode of infection was associated with diarrhea if at least 1 day met the definition for a day with diarrhea during the infection episode or within 1 week of the beginning or end of the episode.

We included children in the epidemiologic analysis if they had been monitored for at least 6 months and at least 24 stool specimens had been submitted for analysis. All statistics were calculated with SAS for Windows, version 8.0. We tested for seasonality and trends associated with diarrhea and infection order by Poisson regression analyses in SAS Proc Genmod (SAS, Cary, NC), incorporating generalized estimating equations to account for correlation between multiple observations from the same person. We assumed an exchangeable correlation structure. Relative risks were assessed for significance by the chi-square test. All statistical results were evaluated at the 0.05 level of significance.

## Results

Of 533 children originally recruited for the cohorts, 368 children (201 [55%] boys) met our inclusion criteria. The 165 excluded children were comparable in age and sex distribution with study children; for 25 children, no stool specimen was submitted, while <6 months of surveillance was completed for the others. At the time of entry into the study, 256 children (70%) were <1 year, 45 (12%) were 1–4 years, and 67 (18%) were 5–11 years of age. The 368 children contributed a total of 889 child-years of surveillance data; nearly half the data were from children <2 years of age. Children were followed for a mean of 2.4 years and had an average of 1.95 episodes of diarrhea/child-year of follow-up. The highest incidence of diarrhea, 3.3 episodes/child-year, was recorded in children 12–23 months old; after the age of 3 years the incidence of diarrhea declined to <1 episode/child-year. The median duration of diarrheal episodes was 2 days (range 1–27). A total of 44,042 stool specimens were screened for coccidian parasites, a median of 124 stools/child (range 24–227); 897 (2%) of the stool specimens were collected during diarrheal episodes.

Children had an average of 0.20 episodes of cyclosporiasis/year and 0.22 episodes of cryptosporidiosis/year of follow-up (Table 1). Of the 368 children, 123 (33%) had at least one detected episode of *Cyclospora* infection, 30 children had two infections, and 10 children had >3 infections. A total of 143 children (39%) had at least one *Cryptosporidium* infection; 34 children had two infections, and 9 had >3 infections. Rates varied by age: the incidence of cryptosporidiosis peaked at 1 year and then fell sharply, but cyclosporiasis incidence remained fairly constant during the 1- to 9-year age period. For the 189 children who were enrolled in the study before the age of 3 months, the mean age at first infection was older for cyclosporiasis than for cryptosporidiosis (1.69 versus 1.36 years; p<0.01). After an initial episode of cyclosporiasis*,* the likelihood of diarrhea decreased significantly (p=0.049) with each subsequent infection (Table 2). For cryptosporidiosis, this trend was less consistent and did not reach statistical significance.

**Table 1:** Incidence of coccidial infections and association with diarrhea by age group, Peru, February 1995 to December 1998

Age^a^ (years)	Child-years of follow-up	Cryptosporidiosis			Cyclosporiasis		
		Infections	Infections/ child-year	With diarrhea n (%)	Infections	Infections/ child-year	With diarrhea n (%)
<1	230.6	47	0.20	20 (43)	16	0.07	4 (25)
1	192.0	80	0.42	32 (40)	40	0.21	11 (28)
2–4	243.2	58	0.24	10 (17)	67	0.28	18 (27)
5–9	170.3	10	0.06	2 (20)	47	0.28	6 (13)
10–12	53.5	1	0.02	0 (0)	4	0.07	1 (25)
<12	889.5	196	0.22	64 (33)	174	0.20	40 (23)

^a^Age on the first day of parasite detection.

**Table 2:** Coccidial infections and their association with diarrhea in Peruvian children, February 1995 to December 1998

Infection order	*Cryptosporidium* episodes			*Cyclospora* episodes		
	Total	With diarrhea^a^	Without diarrhea^a^	Total	With diarrhea^b^	Without diarrhea^b^
First	143	52 (36)	91 (64)	123	33 (27)	90 (73)
Second	43	9 (21)	34 (79)	38	6 (19)	32 (81)
Third	9	3 (33)	6 (67)	10	1 (10)	9 (90)
Fourth	1	0 (0)	1 (100)	2	0 (0)	2 (100)
Fifth	0			1	0 (0)	1 (100)
All episodes	196	64 (33)	132 (67)	174	40 (23)	134 (77)

^a^Test for trend in proportion with diarrhea by infection order, p=0.17. ^b^Test for trend in proportion with diarrhea by infection order, p=0.049.

In a regression analysis in which data were controlled for concurrent cyclosporiasis, the expected mean duration of oocyst shedding was longer for cryptosporidiosis associated with diarrhea than for cryptosporidiosis not associated with diarrhea (expected mean 9.4 versus 4.8 days; p=0.0002). In the analogous regression analysis for concurrent cryptosporidiosis, a similar relationship was found for *Cyclospora cayetanensis* shedding with and without diarrhea (15.7 days versus 6.2 days; p=0.004). Diarrheal episodes associated with cryptosporidiosis*,* but not cyclosporiasis, lasted longer than diarrheal episodes not associated with coccidian parasites (expected mean duration 4.67 days for cryptosporidiosis versus 2.55 days with no coccidia; p<0001; mean 2.96 days for cyclosporiasis versus 2.55 days with no coccidia; p=0.35).

Both parasitic infections were more frequent during December to May than June to November, but the effect was more marked for cyclosporiasis (relative risk [RR] 3.3; p<0.0001) than for cryptosporidiosis (RR 1.9; p<0.0001). After data were adjusted for seasonality and age, the risk for cyclosporiasis or cryptosporidiosis did not differ by household water supply at the time of entry into the study (Table 3). Using a field rather than a toilet or latrine for defecation was associated with a higher risk of cryptosporidiosis but not cyclosporiasis. No association with exposure to animals could be demonstrated for cryptosporidiosis, but cyclosporiasis was more common in children in households with avians, guinea pigs, rabbits, or any other domestic animal.

**Table 3:** Associations^a^ of environmental exposures and infection with coccidian parasites in 368 Peruvian children, February 1995 to December 1998

	Child-years of follow-up	Cyclosporiasis				Cryptosporidiosis			
		No. of episodes	Incidence density^b^	RR (95% CI)^c^	p	No. of episodes	Incidence density^b^	RR (95% CI)	p
Water truck	45.2	11	0.24	0.85 (0.58, 1.26)	0.2	7	0.22	1.01 (0.77, 1.33)	0.94
Water source outside house	268.7	44	0.16	1.01 (0.56, 1.82)	0.98	65	0.24	0.84 (0.49, 1.45)	0.54
Water connection in house	548.1	114	0.21	Referent		119	0.16	Referent	
Defecates in field	298.7	36	0.22	1.00 (0.69, 1.47)	0.99	47	0.29	1.26 (0.97, 1.67)	0.08
Latrine	163.7	54	0.18	0.86 (0.69, 1.47)	0.45	59	0.20	0.96 (0.71, 1.32)	0.85
Flush toilet	380.7	78	0.21	Referent		84	0.22	Referent	
Any animal	665.9	147	0.22	1.72 (1.08, 2.72)	0.02	140	0.21	0.97 (0.74, 1.26)	0.82
No animals	223.6	27	0.12	Referent		56	0.25	Referent	
Chickens	357.7	76	0.21	1.10 (0.81, 1.49)	0.52	75	0.21	1.0 (0.77, 1.29)	0.99
No chickens	531.9	98	0.18	Referent		121	0.23	Referent	
Ducks	245.0	52	0.21	1.07 (0.78, 1.48)	0.64	47	0.19	0.89 (0.67, 1.21)	0.47
No ducks	644.5	122	0.19	Referent		149	0.23	Referent	
Any avian^d^	477.6	108	0.23	1.34 (0.96, 1.87)	0.08	97	0.20	0.91 (0.71, 1.17)	0.48
No avians	412.0	66	0.16	Referent		99	0.24	Referent	
Dog	316.9	68	0.22	1.19 (0.88, 1.59)	0.26	70	0.22	0.93 (0.71, 1.21)	0.58
No dog	572.6	106	0.19	Referent		126	0.22	Referent	
Guinea pig	37.5	12	0.32	1.56 (0.99, 2.44)	0.05	10	0.27	1.63 (0.89, 2.99)	0.11
No guinea pig	852.0	162	0.19	Referent		186	0.22	Referent	
Rabbit	49.6	20	0.40	2.13 (1.23, 3.69)	0.007	11	0.22	1.05 (0.56, 2.0)	0.87
No rabbit	840.0	154	0.18	Referent		185	0.22	Referent	

^a^The analyses were adjusted for seasonality and age of child. ^b^Episodes of infection per child-year of follow-up. ^c^RR=relative risk; CI=95% confidence intervals. ^d^Any avian=one or more of the following birds: chickens, ducks, turkeys, pigeons, parrots, or parakeets.

## Discussion

Our analysis confirms that cryptosporidiosis and cyclosporiasis are common infections in this community, with distinct age-related patterns of occurrence. As noted (4,15), cyclosporiasis affected cohort children at later ages than cryptosporidiosis. The reasons for this epidemiologic pattern are not clear. One possible explanation might be that early infections afford less effective immunity for *Cyclospora cayetanensis* than for *Cryptosporidium parvum*. However, our data appear to contradict this hypothesis: first episodes of cyclosporiasis, but not cryptosporidiosis, protect against later symptomatic infection with the same organism. The development of better laboratory tools will be essential to elucidate the immune mechanisms involved. Another possibility is that the differences in age-specific incidence are related to predominant modes of exposure. This hypothesis is consistent with the assumption that *Cyclospora cayetanensis* is usually transmitted by exposure to contaminated environmental sources, from which young infants are usually relatively protected, while cryptosporidiosis can be transmitted by many routes, including from one toddler to another.

The ability of cryptosporidiosis to cause multiple symptomatic episodes may also be related to genetic heterogeneity. In a previous study of specimens from the same cohort, most cryptosporidiosis in this shantytown was caused by the *Cryptosporidium parvum* human genotype. However, children also had infections with *C. parvum* bovine and dog genotypes, *C. meleagridis*, and *C. felis* (8). Heterologous immunity may be less effective than homologous immunity. Although some polymorphism has been demonstrated in *Cyclospora cayetanensis* isolates (19), genetic studies are still in the early stages, and, to date, this parasite appears to be less heterogeneous than *Cryptosporidium* species. Nevertheless, immunity to both organisms occurs in this highly endemic setting, since immunocompetent adults rarely experience symptomatic infections (4).

For both coccidia, we found a high proportion of infections without diarrhea: 67% of cryptosporidiosis and 77% of cyclosporiasis episodes were not associated with diarrhea. These results differ from those reported in studies with different designs and reflect our methods, which aimed to detect as many coccidial infections as possible, independent of symptoms. Each child had a stool specimen screened nearly every week, so that 98% of the stool specimens were not collected during diarrheal episodes. Nevertheless, serologic data suggest that even intensive stool surveillance may miss a substantial proportion of cryptosporidiosis episodes (20). Data from the same Peruvian community showed that cryptosporidiosis without diarrhea had a substantial effect on childhood growth (21,22); because such infections may have long-term sequelae it is somewhat misleading to call them asymptomatic.

*Cryptosporidium* species infect a wide range of mammalian hosts and can be zoonotic infections (6), while *Cyclospora cayetanensis* has never been convincingly demonstrated to infect a nonhuman host (9–11). However, the animals most commonly associated with zoonotic *C. parvum* were rare in this urban setting: no families had calves, only one family had goats and lambs, and six families had adult sheep. This finding may explain why we could not show an association of cryptosporidiosis with animal exposure. Children in households with animals, especially birds, guinea pigs, and rabbits, appeared to be at higher risk of cyclosporiasis. The finding of an association with avians is consistent with results of a *C. cayetanensis* case-control study in Guatemala (14), but this association is still unexplained. Possibly the presence of domestic animals is a marker for some other unmeasured risk factor. Cyclosporiasis appears to be much more common in Lima than in the mountains of Peru (Asociación Benéfica PRISMA, Lima, Peru, unpub. data), and most residents of the study community migrated from the mountains to Lima. Raising domestic animals, especially poultry, may be more common among recent rural migrants with less exposure and therefore higher susceptibility to *C. cayetanensis*.

Our exposure data were collected at the beginning of longitudinal surveillance, and the time that elapsed between their collection and the occurrence of an infection could have been as long as several years, which may have decreased our ability to detect associations. Studies specifically designed to identify risk factors close to the time of an infection and use of molecular techniques to distinguish genotypes or strains may help clarify some of these issues.

As more longitudinal data become available for these organisms, we are gaining a clearer picture of the overall epidemiology of cyclosporiasis and cryptosporidiosis in endemic settings. Both organisms cause a spectrum of disease, from apparently asymptomatic infection to prolonged episodes of diarrhea that may have a profound effect on a child’s well-being. Our findings for cryptosporidiosis are consistent with the view that the organism infects children very early in life and has multiple routes of transmission, but mysteries remain concerning the cycle that maintains cyclosporiasis as an endemic infection. In-depth study of the transmission of *C. cayetanensis* will be key to designing effective strategies for intervention.
